# A Multidimensional Assessment of Sleep Disorders in Long COVID Using the Alliance Sleep Questionnaire

**DOI:** 10.3390/healthcare13202611

**Published:** 2025-10-16

**Authors:** Alina Wilson, Giorgio Camillo Ricciardiello Mejia, Sara Lomba, Linda N. Geng, Sanjay Malunjkar, Hector Bonilla, Oliver Sum-Ping

**Affiliations:** 1 Department of Psychiatry and Behavioral Sciences, Stanford University School of Medicine, Stanford, CA 94305, USAgiocrm@stanford.edu (G.C.R.M.);; 2 Department of Medicine, Stanford University School of Medicine, Stanford, CA 94305, USA; 3 Research Technology, Stanford University School of Medicine, Stanford, CA 94305, USA

**Keywords:** long COVID, sleep disturbances, alliance sleep questionnaire, principal component analysis, insomnia

## Abstract

**Background/Objectives:** Sleep disturbances are recognized as a common feature of Long COVID but detailed investigation into the specific nature of these sleep symptoms remain limited. This study analyzes comprehensive sleep questionnaire data from a Long COVID clinic to better characterize the nature and prevalence of sleep complaints in this population. **Methods**: We conducted a cross-sectional analysis of 200 adults referred to the Stanford Long COVID Clinic. Patients completed an intake questionnaire including three sleep-related items (unrefreshing sleep, insomnia, daytime sleepiness) rated on a 0–5 Likert scale. Additionally, patients completed the Alliance Sleep Questionnaire (ASQ), incorporating the Insomnia Severity Index, Epworth Sleepiness Scale, reduced Morningness–Eveningness Questionnaire, and modules for parasomnia, restless legs, and breathing symptoms. We calculated the prevalence of six sleep symptom domains. Standardized symptom data were analyzed using principal component analysis (PCA) and K-means clustering (k = 2) to explore latent phenotypes and used logistic regression to assess associations between demographic and clinical variables and each sleep complaint. **Results**: Sleep-related breathing complaints affected 57.5% of participants, insomnia 42.5%, and excessive daytime sleepiness 28.5%. Parallel analysis supported a nine-factor structure explaining ~90% of variance, with varimax rotation yielding interpretable domains such as insomnia/unrefreshing sleep, fatigue/post-exertional malaise, parasomnias, and respiratory symptoms. Gaussian mixture modeling favored a two-cluster solution (n = 94 and n = 106); one cluster represented a higher-burden phenotype characterized by greater BMI, insomnia, daytime sleepiness, gastrointestinal symptoms, and parasomnias. Logistic models using factor scores predicted insomnia with high accuracy (AUC = 0.90), EDS moderately well (AUC = 0.81), but extreme chronotype poorly (AUC = 0.39). In adjusted models, hospitalization during acute COVID-19 was significantly associated with insomnia (OR 4.41; 95% CI 1.27–15.36). Participants identifying as multiracial had higher odds of insomnia (OR 3.22; 95% CI 1.00–10.34), though this narrowly missed statistical significance. No other predictors were significant. **Conclusions**: Sleep disturbances are frequent and diverse in Long COVID. Factor analysis showed overlapping domains, while clustering identified a higher-burden phenotype marked by more severe sleep and systemic complaints. Symptom-based screening may help target those at greatest risk.

## 1. Introduction

A substantial subset of people infected with severe acute respiratory syndrome coronavirus 2 (SARS-CoV-2) can experience long-term symptoms beyond the acute phase of infection, known as Long COVID (also known as long-haul COVID and Post-Acute Sequelae of SARS-CoV-2 infection (PASC) [1,2,3,4]. Long COVID has emerged as one of the most enduring challenges caused by the SARS-CoV-2 pandemic virus. Few countries have implemented surveillance systems to assess the population-level burden of Long COVID, but the available data converges around a prevalence of 6% to 7% in adults and less than 1.5% in children [5,6,7,8,9,10]. Experts estimate the cumulative global incidence of Long COVID is 400 million individuals with an annual economic impact of 1 trillion dollars [11].

Long COVID has been associated with over 200 symptoms, affecting multiple organ systems throughout the body [4,12], including the cardiovascular [13], nervous [14,15,16,17], the endocrine [18,19,20], the immune [21,22], the reproductive [23], and the gastrointestinal systems [24]. Clinically, Long COVID symptoms can be variable but often manifest as unremitting fatigue, post-exertional malaise, cognitive impairment, and autonomic dysfunction, alongside other less common symptoms [25,26,27]. Among the myriad of long COVID symptoms, sleep disturbances have emerged as a significant yet understudied concern.

Sleep is a complex phenomenon with significant impacts on overall health. Consistent poor sleep duration or quality may increase the risk of many chronic health problems, including heart disease, stroke, obesity, diabetes, kidney disease, and depression [28]. The significance of addressing sleep issues is underscored by the American Heart Association, which, as of 2020, has recognized sleep as the eighth metric of heart health, elevating its importance to that of traditional cardiovascular risk factors such as nicotine exposure, cholesterol levels, and blood pressure [29]. The International Classification of Sleep Disorders Third Edition Text Revision categorizes sleep disorders into six main groups: insomnia disorders, sleep-related breathing disorders, central disorders of hypersomnolence, circadian rhythm sleep–wake disorders, parasomnias, and sleep-related movement disorders [30]. Sleep disturbances are extremely common [31]. Obstructive sleep apnea alone is estimated to affect up to 1 billion people globally, with the majority of cases going undiagnosed and untreated [31,32,33]. Similarly, insomnia is a widespread issue, impacting over 10% of the adult population [32,34].

Although sleep is crucial and disruptions can have severe consequences, the existing literature on Long COVID-related sleep disturbances offers limited and inconsistent prevalence data. A 2022 meta-analysis of 63 studies found that sleep disorders affected approximately 24% of patients who had experienced COVID-19 between 3 and 6 months prior, 29% of those 6 to 9 months post-infection, and 30% more than 12 months after infection. Reported figures in individual studies varied significantly, with one online study reporting that 78.58% of subjects with long COVID had sleep disturbances [35]. The Researching COVID to Enhance Recovery (RECOVER-Adult) study, a prospective, observational cohort study, reported that sleep disturbances were present in 32% of the cases. Snoring or sleep apnea in particular was present in 38% of the cases and was also identified as one of the 11 most distinguishing symptoms of those with COVID infection compared to those without [36].

Furthermore, while the meta-analysis identified an upward trend in the prevalence of sleep disorders over time following infection, some studies have observed the opposite pattern [37].

Although awareness of sleep disturbances impacting long COVID patients has increased, the literature remains limited in exploring specific sleep complaints and their associations in long COVID populations [12]. Much of the available literature focuses on overall sleep quality rather than delving into more granular phenotypes of sleep complaints. The data assessing long COVID patient sleep disturbances using specific, validated subjective sleep scales are also limited [13]. While a handful of studies employ scales such as the Pittsburgh Sleep Quality Index, Epworth Sleepiness Scale, or the Insomnia Severity Index, few studies also investigate sleep complaints, including restless legs syndrome, extreme circadian phenotypes, sleep-related breathing disorders, or parasomnia activity [14]. Furthermore, the literature surrounding Long COVID generally focuses on isolated individual symptoms. Yet, considering that published studies demonstrate that long COVID includes a myriad of symptoms that affect nearly every system in the body, focusing on symptom clusters may aid in pinpointing the underlying pathophysiological mechanisms and better sub-classifying patients [15,38].

Further investigation is needed to investigate how this multisystemic disease affects the body. The purpose of this study is to further investigate sleep symptoms in the setting of long COVID with a detailed analysis of our clinical cohort at an academic medical center and an exploration of factors that may influence the development or exacerbation of sleep symptoms or symptom clusters in these patients.

## 2. Materials and Methods

### 2.1. Study Design and Participants

We conducted a cross-sectional study to understand the relationship between long COVID symptoms and sleep disturbances, utilizing standardized sleep questionnaires, the long COVID standardized clinical assessment, and retrospective chart review of patients in the long COVID Clinic at Stanford Medicine. The study was approved by the Stanford Institutional Review Board

The Stanford Long COVID Clinic is a multidisciplinary program that provides clinical expertise in post-COVID-19 conditions, streamlines data collection and clinical management, and facilitates the integration of research. The long COVID clinic utilizes a referral network of diverse specialties, from internal medicine, neurology, cardiology, psychiatry, pulmonology, rheumatology, otolaryngology, immunology, gastroenterology, and sleep medicine. It serves over 1000 patients with long COVID symptoms. During the period of data collection (5 October 2023, to 2 July 2024) patients were required to meet the following eligibility guidelines to be seen by the clinic: adults over 18 years old, with a positive result for COVID-19 confirmed by reverse transcription-polymerase chain reaction for SARS-CoV-2, nucleic acid amplification tests (NAAT), antibodies before vaccination, or antigen test, and experiencing persistent symptoms for at least 28 days following SARS-CoV-2 infection [39].

Upon intake, all patients complete a general questionnaire to record their symptoms and COVID-19 history. Three questions on the routine clinic questionnaire addressed sleep disorders. These questions addressed symptoms of unrefreshing sleep, difficulty sleeping/insomnia, and daytime sleepiness and ranked the severity of these symptoms on a Likert Scale of 0 to 5, with 0 indicating the absence of a symptom and 5 indicating the most severe form.

Patients were also asked to complete the Alliance Sleep Questionnaire (ASQ) as part of their clinical evaluation. The ASQ is an online sleep questionnaire developed by a team of domain experts, comprising validated sleep measures and novel questions. The questionnaire utilizes branching logic to guide patients through a comprehensive set of questions covering demographic information, medical history, medications, sleep history, sleep habits, and schedule, as well as symptoms of insomnia, excessive daytime sleepiness, extreme circadian phenotype, restless legs, parasomnia activity, and sleep-related breathing complaints. The ASQ was implemented successfully at Stanford’s Sleep Clinic before its extension to the Long COVID clinic [40]. Data were retrospectively extracted from the Electronic Health Record (EHR) for patients seen in the long COVID clinic.

### 2.2. Variables

For this study, we analyzed data on various demographic and medical history types from the HER and the ASQ.

- Demographic Information: We extracted demographic information (sex at birth, age, Body Mass Index (BMI), race/ethnicity).
- Vaccination Status: We considered vaccination, defining it as people who received at least two doses of the SARS-CoV-2 vaccine, regardless of whether the vaccine was mRNA (Moderna, Cambridge, MA, USA or Pfizer, New York, NY, USA) or a virus vector vaccine (Johnson & Johnson, New Brunswick, NJ, USA or AstraZeneca/Oxford, Cambridge, UK). There is a well-documented benefit of SARS-CoV-2 vaccination, such as on severity, emergency room visits, hospitalization, ICU admission, mortality, cardiovascular events, and Long COVID [41,42,43]. We planned to evaluate in our study population whether vaccination has any impact on sleep disorders; however, this population was highly vaccinated (173/200, 86.5%), and we consider the difference sufficient to conduct this kind of analysis.
- Sleep Complaint History: We defined individuals with “sleep complaints before SARS-CoV2 infection” as those who had a recorded history of any sleep complaint in their medical charts following a detailed manual chart review performed by one of four reviewers.
- COVID-19 History: We collected information on days from the initial COVID-19 infection and acute COVID-19 infection treatment (hospitalized/ambulatory) until completion of the ASQ. When assessing the number of hospitalized patients for this study, we considered each instance of SARS-CoV-2 acute infection for individuals who experienced COVID-19 multiple times.

### 2.3. Sleep Symptoms

Patients in the Long COVID Clinic were provided the ASQ as part of their usual care and a retrospective analysis was performed on completed questionnaires. A standardized tool developed through consensus among industry leaders, the ASQ offers a comprehensive assessment of the patient’s overall health, including sleep issues, and employs branching-logic algorithms to optimize the patient’s time utilization [40]. The ASQ collects self-reported sleep complaints and medical history. Embedded within the ASQ are the Insomnia Severity Index (ISI) [44], Epworth Sleepiness Scale (ESS) [45], and the reduced Morningness-Eveningness Questionnaire (rMEQ) [46]. From the ASQ, we extracted the following: the ISI, the ESS, the rMEQ, and self-reported sleep complaints related to parasomnia, restless legs, and sleep-related breathing.

### 2.4. Statistical Analysis

The ASQ comprises up to 16 questions to assess the likelihood of restless legs syndrome (RLS), yielding a unique RLS probability score specific to the ASQ. Scoring ranges from unlikely (possibly in the past) to possible and likely. Based on expert opinion, individuals scored as “likely” were categorized as having a complaint related to RLS. The ASQ includes a parasomnia module in which patients can self-report the frequency of five specific sleep-related behaviors (sleepwalking, sleep eating, dream enactment, violent behavior during sleep, sex with no memory). Any self-reported activity in these categories was considered indicative of parasomnia activity.

Additionally, sleep-related breathing complaints included symptom screening for loud snoring, snorting and gasping, and apneic events, where the presence of any of these indicated a sleep-related breathing complaint. Extreme circadian phenotype was defined as a score of 3–7 (definite evening type) or 22–25 (definite morning type) on the rMEQ. Extreme circadian rhythms were determined by the rMEQ score, with scores within 3 and 7, or greater than 22 as extreme. Excessive daytime sleepiness was defined using an ESS score of 10 or above. Responders with a score of 15 or higher on the ISI were considered to have the complaint of insomnia.

To summarize symptom structure, we applied Horn’s parallel analysis to determine the number of components to retain, followed by PCA with varimax rotation for interpretability [47]. We computed rotated factor scores by least-squares projection of standardized features onto the rotated loadings. To make factors directionally interpretable, we aligned factor signs to a severity anchor (binarized self-reported groups given by ISI, ESS, rMEQ, and FOSQ) so that higher factor values correspond to worse symptoms.

We fit Gaussian mixture models (GMMs) [48] with full covariance on the factor space and selected the number of components k  ∈  {1,…,6} by Bayesian Information Criterion (BIC) [49]. For each k > 1, we computed silhouette scores; when the selected model had k > 1, we also assessed cluster stability via K-means adjusted Rand index (ARI) across 20 runs [50]. Labels and sizes for the selected solution were recorded. Clustering is treated as exploratory given expected heterogeneity.

For visualization only, we embedded factor scores into 2D with UMAP (Euclidean metric; neighbors = 15; minimum distance = 0.10) and plotted cluster overlays with confidence ellipse [51].

We reported silhouette, Calinski–Harabasz, and Davies–Bouldin indices. Pairwise separation was further summarized with centroid distances and Bhattacharyya coefficients. Between-cluster comparisons used χ^2^ or Fisher’s exact (categorical), independent-samples *t*-tests or Mann–Whitney U (continuous), and Kruskal–Wallis where appropriate.

To evaluate clinical relevance of the latent factors, we used the predefined endpoints (insomnia, EDS, extreme chronotype). Factor scores were correlated with the continuous scales ISI, ESS, PSQI, and rMEQ (plus a composite symptom index, CSI) using Spearman correlations. Logistic regression models with factor scores as predictors were evaluated with five-fold stratified cross-validation, reporting mean AUC, average precision, and Brier score across folds. Sample size adequacy for logistic regression endpoints was assessed using events-per-variable (EPV) metrics [52].

Multivariable regression models were implemented to assess associations between potential predictors (e.g., demographic and clinical variables) and key outcomes (e.g., sleep disturbances and symptom severity). Both logistic and linear regression models were fitted, depending on the outcome type (binary or continuous). Adjusted and unadjusted models were constructed to isolate the effects of covariates and identify independent risk factors.

Analyses were performed in Python 3.9.18 using scikit-learn 1.3.0 (PCA, GMM, K-means, metrics), SciPy 1.11.3 (hypothesis tests), UMAP version 0.5.9, and statsmodels (version 0.14.4) for FDR procedures. Code for the full analysis pipeline is available in a public repository [53].

## 3. Results

From 5 October 2023 to 2 July 2024, the ASQ was administered to 604 patients, with 200 completing the questionnaire. Table 1 provides an overview of the demographic characteristics of the long COVID analysis study cohort of these 200 patients.

The average age of the patients was 46.5, ranging from 21 to 77 years. The mean duration of symptoms was 770 days (from the time of initial SARS-CoV-2 infection to the completion of ASQ), ranging from 64 to 1468 days. The mean BMI of the study population was 26.78 (6.59). Of the 200 clinic patients in this study, 134 (67.0%) were female, and 66 (33%) were male. One hundred and thirty-nine (69.5%) of patients identified as White, six as Black (3.00%), thirty-four as Asian (17.0%), three as American Indian/Native American (1.50), one as Pacific Islander (0.50%), and seventeen as multiple races (8.50%). Further, twenty-two patients were identified as Hispanic (11.00%). Most patients completed some form of higher education (57.50%). Most (93.00%) did not require hospitalization during their initial acute COVID-19 infection, and 86.50% had received the SARS-CoV-2 vaccination. A thorough review of the patient’s medical records revealed that only a minority (7.00%) had a documented history of sleep complaints. According to the clinic assessment, 169 patients (84.50%) reported functional impairment, categorized as function status III, IV, or V. Furthermore, 75 (37.50%) patients exhibited significantly compromised well-being, as indicated by a functional status of IV or V [39].

### 3.1. Prevalence of Specific Long COVID Sleep Complaints

**Figure 1:**
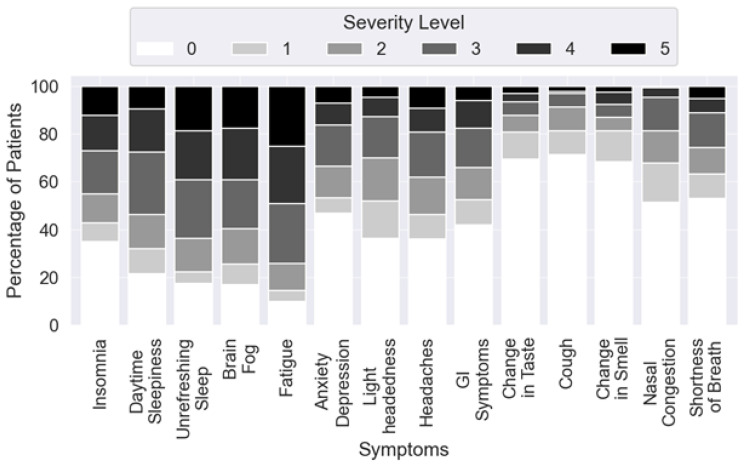
Subjective responses collected from the patients at baseline. All questions are presented as a six-point Likert scale, with zero meaning no symptoms and five as most severe.

Figure 1 shows subjective symptom severity ratings from 200 patients at the time of intake into the Stanford Long COVID Clinic. This was obtained through a general intake survey, which included questions about sleep and other symptoms. Each symptom’s severity was scored on a five-point scale, from 0 (no symptoms) to 5 (most severe). The distribution of responses suggests that while some symptoms were reported at higher severity levels (e.g., fatigue, anxiety, and insomnia), others showed more variability in perceived intensity across the cohort.

We also investigated the prevalence of six categories of sleep complaints through the ASQ: insomnia, excessive daytime sleepiness, sleep-related breathing symptoms, restless legs, parasomnia activity, and extreme circadian phenotype (Table 2).

### 3.2. Long COVID Symptom Clusters

Parallel analysis retained 9 components (observed eigenvalues exceeded the random–data mean for components 1–9; crossover occurred at component 10). The cumulative explained variance of the first 1–9 components was 0.43, 0.54, 0.65, 0.74, 0.81, 0.86, 0.90, 0.95, 1.00, respectively (Supplementary Figure S1A,B). Gaussian mixture modeling on the factor scores minimized BIC at k = 2 (Supplementary Figure S1C), yielding cluster sizes n = 94 (47%) and n = 106 (53%). Internal validity metrics for this two-component solution were: silhouette = 0.174, Calinski–Harabasz = 50.024, and Davies–Bouldin = 1.797. In the UMAP embedding, the between-cluster centroid distance = 2.115 and Bhattacharyya coefficient = 0.291 (Supplementary Figure S2).

Across 200 participants, there were 85 cases of insomnia (EPV = 9.4), 57 cases of excessive daytime sleepiness (EPV = 6.3), and 30 cases of extreme chronotype (EPV = 3.3) (Supplementary Table S1). Performance across the five-fold stratified cross-validated logistic models with factor scores as predictors was for: (i) insomnia (ISI ≥ 15)—AUC 0.896, AP 0.890, Brier 0.129; (ii) excessive daytime sleepiness (ESS ≥ 10)—AUC 0.810, AP 0.652, Brier 0.152; (iii) extreme chronotype (rMEQ ≤ 7 or ≥22)—AUC 0.386, AP 0.185, Brier 0.141.

We examined how symptom factors related to standard sleep questionnaires. The strongest link was between Factor 1 (insomnia/unrefreshing sleep) and the ISI, with a correlation of 0.73 (*p* < 10^−33^). Additional associations included Factor 3 with ISI (ρ = 0.58, *p* < 10^−19^) and Factor 9 with both ISI (ρ = 0.48, *p* < 10^−12^) and daytime sleepiness (ESS, ρ = 0.49, *p* < 10^−13^), while several other factors showed more modest but still significant relationships with sleep quality (PSQI) and rMEQ (Supplementary Table S2).

**Figure 2:**
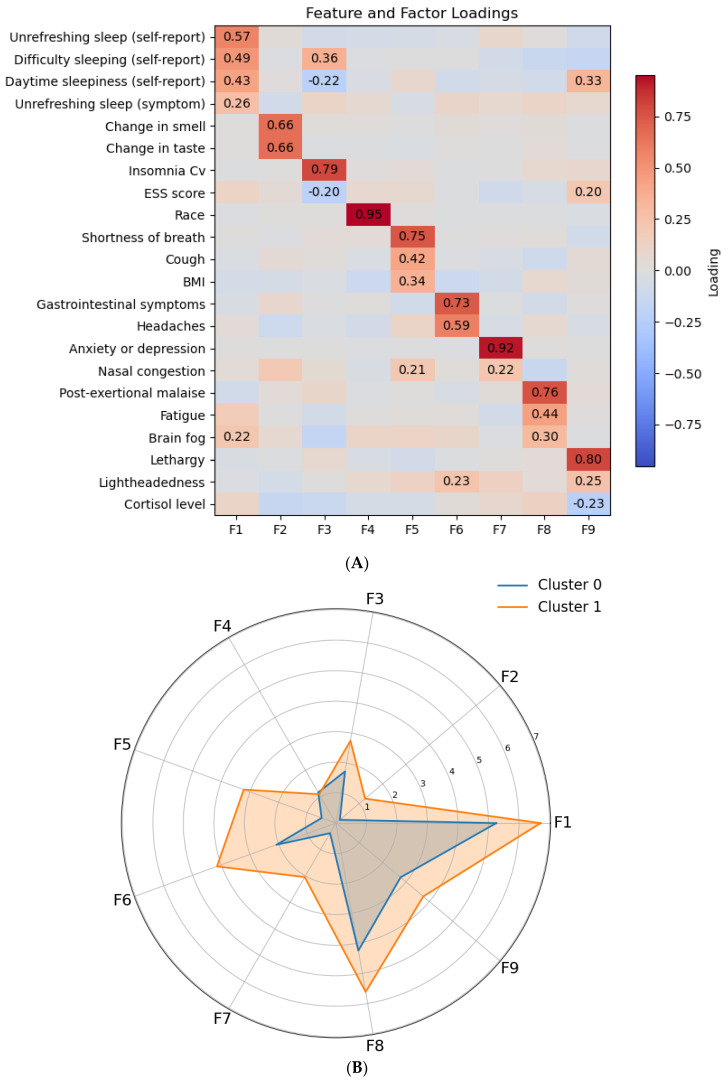
Latent factor structure and cluster profiles. (**A**) Heatmap of rotated factor loadings for nine retained components. For clarity, only variables meeting a minimum absolute loading threshold of 0.24 are shown. Loading matrix is presented in Supplementary Table S3. Selected symptoms (rows) align with factors (columns). (**B**) Radar plot of average factor scores for the two GMM clusters (Cluster 0 = blue, Cluster 1 = orange). Cluster 1 shows relatively higher insomnia- and fatigue-related factor scores, while both clusters share overlapping symptom patterns overall. Insomnia Cv (Long Covid Clinic).

Varimax-rotated factor loadings for the nine retained components are shown in Figure 2A. Clear and distinct domains emerged (Figure 2B). Factor 1 (F1) captured unrefreshed and difficulty sleeping complaints, with strong contributions from self-reported unrefreshing sleep, difficulty sleeping, and daytime sleepiness. Factor 2 (F2) reflected sensory changes, with high loadings for changes in smell and taste. Factor 3 (F3) captures the insomnia severity from the Long COVID Clinic questionnaire and the ESS. Factor 5 (F5) captures respiratory complaints. Factor 6 (F6) linked GI symptoms, headaches and lightheadedness. Factor 7 (F7) was strongly driven by anxiety or depression. Factor 8 (F8) reflected post-exertional malaise and fatigue-related complaints, and brain fog with negative associations on difficulty sleeping and daytime sleeping. Finally, Factor 9 (F9) is mainly driven by lethargy and lightheadedness with weaker contributions from cortisol levels.

Between the two BIC-selected clusters (Cluster 0: n = 95; Cluster 1: n = 105), several demographic and clinical features differed significantly. Cluster 1 had higher BMI (*p* = 1.2 × 10^−4^), higher ISI (*p* = 0.017) and ESS scores (*p* = 0.030), and lower FOSQ (*p* = 0.009). The MAP score was more negative in Cluster 1 (*p* = 0.022). Sleep-related complaints were enriched in Cluster 1, including snoring, breathing stops at night, irregular schedules, parasomnias, RLS, unrefreshing sleep, and daytime sleepiness (*p* ≤ 0.037). Gastrointestinal symptoms and post-exertional malaise were also more frequent, while vaccination status showed modest differences (*p* = 0.033; Supplementary Table S4). Results are presented descriptively without correction for multiple comparisons.

### 3.3. Predictors of Sleep Complaints in Long COVID Patients

The logistic regression models of which factors were associated with the six sleep complaints of interest: insomnia, excessive daytime sleepiness, sleep-related breathing symptoms, parasomnia activity, restless legs, and extreme circadian phenotype were not significant associated with Long COVID (Table 3). However, individuals identifying as Multiracial had 3.2 times greater odds of reporting insomnia compared to those who identified as White. In addition, hospitalized participants during their acute COVID-19 infection had 4.4 times the odds of experiencing insomnia compared to non-hospitalized individuals.

## 4. Discussion

In this study, we analyzed detailed sleep assessments from 200 patients with Long COVID using ASQ as an assessment tool and the Long Covid Sleep Questionnaire. Self-reported symptoms from Stanford Long COVID clinic suggest that sleep disorders in the context of Long COVID are prevalent, with 87% reporting at least one sleep complaint (unrefreshing sleep, insomnia, and lethargy) through the general intake screening questionnaire. This number surpasses many previously reported figures on the percentage of long COVID patients experiencing sleep disturbances. For instance, researchers in the Cleveland Clinic found that 41.3% of patients had moderate to severe sleep disturbance [54]. Generally, studies have estimated that 34–50% of long COVID patients experience sleep disturbances [55,56]. Nonetheless, it is notable that only 7.00% of our cohort reported sleep complaints before their COVID-19 infection. Therefore, over ten times as many patients reported sleep complaints after meeting clinical criteria for long COVID, reaffirming that sleep disturbances represent a critical issue in the long COVID population.

More granular sleep data collected through the ASQ suggest that the presence of sleep symptoms is highly variable. Factor analysis revealed a multidimensional symptom structure spanning insomnia/unrefreshing sleep, fatigue/post-exertional malaise, parasomnias, respiratory complaints, and neuropsychiatric symptoms. While Gaussian mixture modeling supported a two-cluster solution, internal validity metrics showed weak separation, suggesting overlapping rather than discrete phenotypes. One cluster, enriched for higher BMI, insomnia, daytime sleepiness, gastrointestinal complaints, parasomnias, and post-exertional malaise, may represent a subgroup with more severe and systemic symptom burden. This lack of distinct clustering reflects the heterogeneous and multidimensional nature of Long COVID in general, though the possibility of contributions from under-measured factors such as pre-COVID morbidities or psychosocial factors remains

Correlations between latent factors and validated sleep scales provided additional evidence of construct validity. For example, the insomnia/unrefreshing sleep factor strongly tracked ISI scores (ρ = 0.73), while other factors showed more modest associations with sleep quality and circadian preference. Predictive modeling further demonstrated that factor scores could distinguish insomnia and excessive daytime sleepiness with good discrimination (AUCs 0.90 and 0.81, respectively), though prediction of extreme chronotype was poor.

This study also sought to contribute to existing literature by diving deeply into specific sleep complaints in the long COVID population. While prior studies have largely focused on single sleep-related symptoms such as insomnia or global sleep quality, our use of the ASQ enabled the identification of more detailed sleep complaints. In our study population, similar to published literature, the most common sleep complaint was insomnia (42.5%); however, the prevalence of excessive daytime sleepiness in this cohort (28.5%) exceeded most previously published rates [56,57] though a higher prevalence (34.5%) of excessive daytime sleepiness has been reported previously in a similar clinic population [58]. Further research is required to investigate the prevalence of excessive daytime sleepiness in this population. Few data regarding sleep symptoms beyond insomnia and excessive daytime sleepiness are available in published literature. In this study, we observed a high prevalence of subjective sleep-related breathing complaints (57.5%), restless legs (15.5%), parasomnia (14.5%), and extreme circadian phenotype (11%) among long COVID patients.

The high prevalence of symptoms found in this cohort underscores the importance of robust sleep symptom screening in Long COVID populations as these sleep symptoms are often under-recognized but nonetheless impairing. Clinicians are advised to employ broad and detailed sleep assessments and not limit screening to insomnia, fatigue or generic sleep disruption. Identification and treatment of these symptoms may yield substantial improvement in quality of life for patients and, in some cases, could serve to help mitigate other Long COVID-related symptoms. On a systems level, these findings support the inclusion of sleep medicine expertise into multidisciplinary Long COVID clinics.

Individuals who reported to be multiracial and those who required hospitalization for acute SARS-CoV-2 infection were far more likely to experience insomnia (OR 3.2 and 4.4, respectively). Otherwise, no significant correlations were observed between age, illness duration, sex, race, and BMI and six particular sleep complaints, including insomnia, excessive daytime sleepiness, sleep-related breathing complaints, parasomnia activity, and extreme circadian phenotypes. Prior investigations have found that factors such as hospitalization status, middle age, Black or Hispanic race, higher BMI, and female sex may be associated with more severe sleep disturbances [54,59,60,61,62]. The investigation of specific risk factors and a suite of sleep disturbance indicators is less common within the literature; this study’s statistical power and lack of even spread of variables, such as hospitalization and vaccination status, could have limited our ability to reveal additional findings.

Our study has several limitations that warrant consideration. Data were drawn from a single center in Northern California, which may limit generalizability. The cohort was predominantly highly educated, whereas individuals with lower educational attainment—who face a disproportionate burden of Long COVID [63,64,65,66]—were underrepresented. Similarly, racial and ethnic minority groups, particularly Black individuals, were underrepresented, reducing the ability to examine disparities. The overall sample size was modest relative to the heterogeneity of symptoms, and referral to a specialized Long COVID clinic likely enriched for patients with more severe presentations. Sleep data were based on self-reports from the ASQ without objective measures such as polysomnography, and the cross-sectional design precludes causal inference. Power was adequate for common endpoints (e.g., insomnia, EDS) but limited for less frequent phenotypes like extreme chronotype, and multiple comparisons raise the possibility of chance associations. Finally, the length of the questionnaire may have introduced selection bias toward patients more engaged with their sleep health. Despite these constraints, the study provides granular evidence that sleep disturbances are common and multifaceted in Long COVID.

This study may have several confounding factors. Given that there is limited pre-COVID-19 infection data for these patients, we cannot claim that the sleep disturbances indicated by study data directly result from their initial SARS-CoV-2 infection, as there may be other unmeasured factors influencing their condition. EHR data describing sleep disorders and symptoms were generally sparse for this population prior to COVID. While this may have been due to a lack of true sleep complaints, it is also possible that pre-existing sleep problems were present but insufficiently documented. The lack of controls, such as a cohort infected with COVID-19 but did not develop long COVID, limits our ability to link the sleep symptoms with Long COVID rather than pandemic-related disruptions in daily routines or other factors. Furthermore, the length of the ASQ may contribute to selection bias. Patients who are invested enough in their sleep to complete a comprehensive sleep survey like the ASQ could be so as a result of a sleep disturbance. On the other hand, the comprehensiveness of the ASQ may have excluded individuals experiencing severe fatigue and brain fog from the study, as their symptoms might have hindered their ability to complete the questionnaire thoroughly. Using a cross-sectional and retrospective design in our study subjects to recall bias and constrains our ability to establish causality. While this study would have been strengthened with more objective sleep data such as polysomnography, only a very limited subset of this cohort had undergone any sleep testing, and this was not included in the analysis.

Furthermore, the overall model performance was low across all sleep complaints, with pseudo-R^2^ values below 0.07 and relatively high Bayesian Information Criterion (BIC) values, indicating that the models explained only a small proportion of the variability in outcomes and may not generalize well. These results suggest that additional, unmeasured factors likely contribute to sleep disturbances in this population, and that the predictive power of our models is limited.

While this research provides cross-sectional data on self-reported symptoms in a small long COVID cohort, it does carry several notable considerations for further research and clinical practice. This research underscores the importance of screening long COVID patients for sleep disorders, given the high prevalence of sleep complaints within this population. Clinicians should ask patients about sleep disorders of all types—not just insomnia and daytime sleepiness—given that this study suggests that other forms of sleep disturbances may also be shared in long COVID patient populations. Future research should aim to include a larger, more diverse, and more representative sample through multicenter studies to ensure the robustness and generalizability of findings regarding self-reported symptoms and long COVID patient populations. Longitudinal follow-up would be helpful to better understand the natural history of sleep issues in long COVID. Given the prevalence and diversity of sleep disturbances affecting long COVID patients, more research is needed on the mechanisms underlying this connection, as well as how to treat a range of sleep disturbances in the context of long COVID.

## 5. Conclusions

Long COVID presents a complex and enduring challenge. This study adds to the nascent literature on Long COVID sleep issues and indicates that a significant proportion of long COVID patients may experience sleep disturbances. Factor analysis highlighted overlapping but clinically interpretable domains. Further research is needed regarding understudied sleep issues in long COVID, including parasomnia activity, restless legs, extreme circadian phenotypes, and sleep-related breathing disturbances. These findings serve as valuable insights for establishing coherent symptom patterns, aiding patient education, managing expectations, and fostering patient understanding of their experiences and potential outcomes. Furthermore, these results lay the groundwork for future research endeavors aimed at comprehensively understanding self-reported long COVID symptoms and elucidating the presentation of symptom clusters.

## Figures and Tables

**Table 1:** Patient characteristics and distribution of Long COVID cohort (N = 200).

Parameter	N (%)	Parameter	N (%)
Age (years) [mean (STD)]	46.5 (16.9)	Functional status ^b^	
Days since infection [mean (STD)]	770 (392.61)	II	23 (11.5%)
BMI (kg/m^2^) [mean (STD)]	26.78 (6.59)	III	94 (4.7%)
Sex ^a^		IV	59 (29.5%)
Males	67 (33.0%)	Did not respond	8 (4.00%)
Females	134 (67.0%)	Sleep Complaints Prior to COVID-19 Infection	
Race		Yes	14 (7.00)
White	139 (69.50%)	No	186 (93.0)
Black	6 (3.00%)	Vaccination Status ^c^	
American Indian/Native American	3 (1.50%)	Vaccinated	173 (86.5%)
Asian	34 (17.0%)	Not Vaccinated	27 (13.5%)
Pacific Islander	1 (0.5%)	Initial COVID-19 Infection Hospitalization Status	
Multiple Races	17 (8.50%)	Hospitalized	14 (7.00%)
Ethnicity		Ambulatory	186 (93.00%)
Hispanic Origin	22 (11.0%)		
Non-Hispanic Origin	178 (89.0%)		
Highest Level of Education Attained			
Elementary School	2 (1.0)		
High School	27 (13.5)		
Associate’s degree	23 (11.5)		
Bachelor’s Degree	63 (32.5)		
Graduate Degree	77 (38.5)		
Did Not Reply	4 (2.0)		

^a^ Patients self-reported their sex assigned at birth. ^b^ A functional status of V indicates a state of being incapacitated and bedridden, while II indicates symptomatic without limitations. ^c^ Vaccinated is defined as receiving two doses of the COVID-19 Vaccine, either Pfizer or Moderna and a booster.

**Table 2:** Prevalence of Self-Reported Sleep Complaints Among Patients in the Long COVID Clinic, Stratified by Sex. Fisher exact test was implemented to test for statistical significance.

Complaint	Long COVID Cohort [N (%)]			
	Male (n = x)	Female (n = x)	Total (n = x)	*p*-Value *
Insomnia ^⧫^	23 (34.8%)	62 (46.3%)	85 (42.5%)	0.48960
Excessive daytime sleepiness ^◼^	15 (54.0%)	42 (31.3%)	57(28.5%)	0.48960
Sleep-related breathing complaint	37 (56.1%)	78 (58.2%)	115 (57.5%)	1
Restless legs	7 (10.6%)	24 (17.9%)	31 (15.5%)	0.48960
Parasomnia activity	9 (13.6%)	20 (14.9%)	29 (14.5%)	1
Extreme circadian phenotype	7 (10.6%)	15 (11.2%)	22 (11.0%)	1

^⧫^ Defined by an Insomnia Severity Index score > 15; ^◼^ Defined by an Epworth Sleepiness Scale score >10; * Corrected *p* value for multiple testing.

**Table 3:** Regression model for the presence of sleep symptoms among individuals. In the Long COVID clinic (N = 200).

Sleep Complaint of Interest [OR (95% CI)]						
Variable	Breathing Symptoms	Excessive Daytime Sleepiness	Extreme Circadian	Insomnia	Parasomnia	Restless Legs
Age	1.216 (0.90, 1.65)	0.768 (0.55, 1.07)	0.737 (0.48, 1.14)	1.029 (0.76, 1.38)	1.086 (0.70, 1.69)	1.006 (0.66, 1.54)
Male	0.967 (0.52, 1.78)	0.531 (0.26, 1.11)	0.899 (0.35, 2.30)	0.761 (0.39, 1.47)	0.836 (0.35, 1.99)	0.547 (0.22, 1.38)
BMI	1.216 (0.86, 1.72)	1.214 (0.88, 1.67)	1.150 (0.69, 1.90)	0.741 (0.54, 1.03)	1.246 (0.84, 1.84)	0.963 (0.65, 1.43)
Vaccinated	0.978 (0.42, 2.28)	0.704 (0.27, 1.84)	1.100 (0.29, 4.21)	0.603 (0.25, 1.47)	1.199 (0.34, 4.19)	0.671 (0.23, 1.94)
Duration ^a^	1.274 (0.88, 1.84)	2.574 (0.86, 7.70)	1.063 (0.80, 1.41)	0.902 (0.73, 1.12)	1.031 (0.82, 1.29)	1.103 (0.84, 1.44)
Hospitalized	1.190 (0.30, 4.65)	0.908 (0.28, 2.96)	2.50 × 10^−9^ (9.74 × 10^−10^, 6.43 × 10^−9^)	4.413 * (1.27, 15.36)	0.618 (0.12, 3.08)	3.151 (0.91, 10.92)
Race (White/Caucasian = reference category)						
Asian	1.250 (0.56, 2.78)	2.111 (0.88, 5.08)	0.405 (0.09, 1.81)	1.584 (0.74, 3.39)	2.071 (0.68, 6.29)	0.494 (0.13, 1.87)
Other ^b^	2.934 (0.56, 15.38)	2.603 (0.75, 9.06)	1.160 (0.14, 9.91)	1.315 (0.30, 5.78)	3.298 (0.79, 13.73)	0.772 (0.21, 2.80)
Multiple Races	1.587 (0.53, 4.76)	0.732 (0.22, 2.44)	1.709 (0.43, 6.75)	3.219 * (1.00, 10.34)	1.047 (0.22, 5.06)	0.207 (0.03, 1.66)

* *p*-value < 0.05 Logistic regression. ^a^ Days since acute infection to date of ASQ completion. ^b^ Includes Black/African Americans and any other race.

## Data Availability

The datasets analyzed during the current study are not publicly available due to patient privacy and ethical restrictions. Aggregate data that support the findings of this study are included in the article. Individual-level data may be considered for sharing by the corresponding author upon reasonable request and with approval from the Stanford University Institutional Review Board.

## Supplementary Materials

The following supporting information can be downloaded at https://www.mdpi.com/article/10.3390/healthcare13202611/s1, Figure S1: Factor retention and mixture selection; Figure S2: UMAP Embedding; Table S1: Sample size adequacy for logistic regression endpoints; Table S2: Factor Scores and Severity Symptoms Correlation; Table S3: Complete rotated factor loading matrix; Table S4: Between-cluster comparisons of demographic and clinical features.

## Abbreviations

The following abbreviations are used in this manuscript:

ASQ	Alliance Sleep Questionnaire
BIC	Bayesian Information Criterion
BMI	Body Mass Index
EHR	Electronic Health Record
ESS	Epworth Sleepiness Scale
FDR	False Discovery Rate
ISI	Insomnia Severity Index
NAAT	Nucleic Acid Amplification Tests
PCA	Principal Component Analysis
PASC	Post-Acute Sequelae of SARS-CoV-2 infection
RLS	Restless Legs Syndrome
rMEQ	reduced Morningness-Eveningness Questionnaire
