# Supplementary material for: A Multidimensional Assessment of Sleep Disorders in Long COVID Using the Alliance Sleep Questionnaire
Source: Healthcare (Basel). 2025 Oct 16;13(20):2611. doi: 10.3390/healthcare13202611 (PMC12563213; doi:10.3390/healthcare13202611)
Supplement: Supplementary file 1 [file healthcare-13-02611-s001.zip › healthcare-3787838-supplementary.pdf]

Supplementary

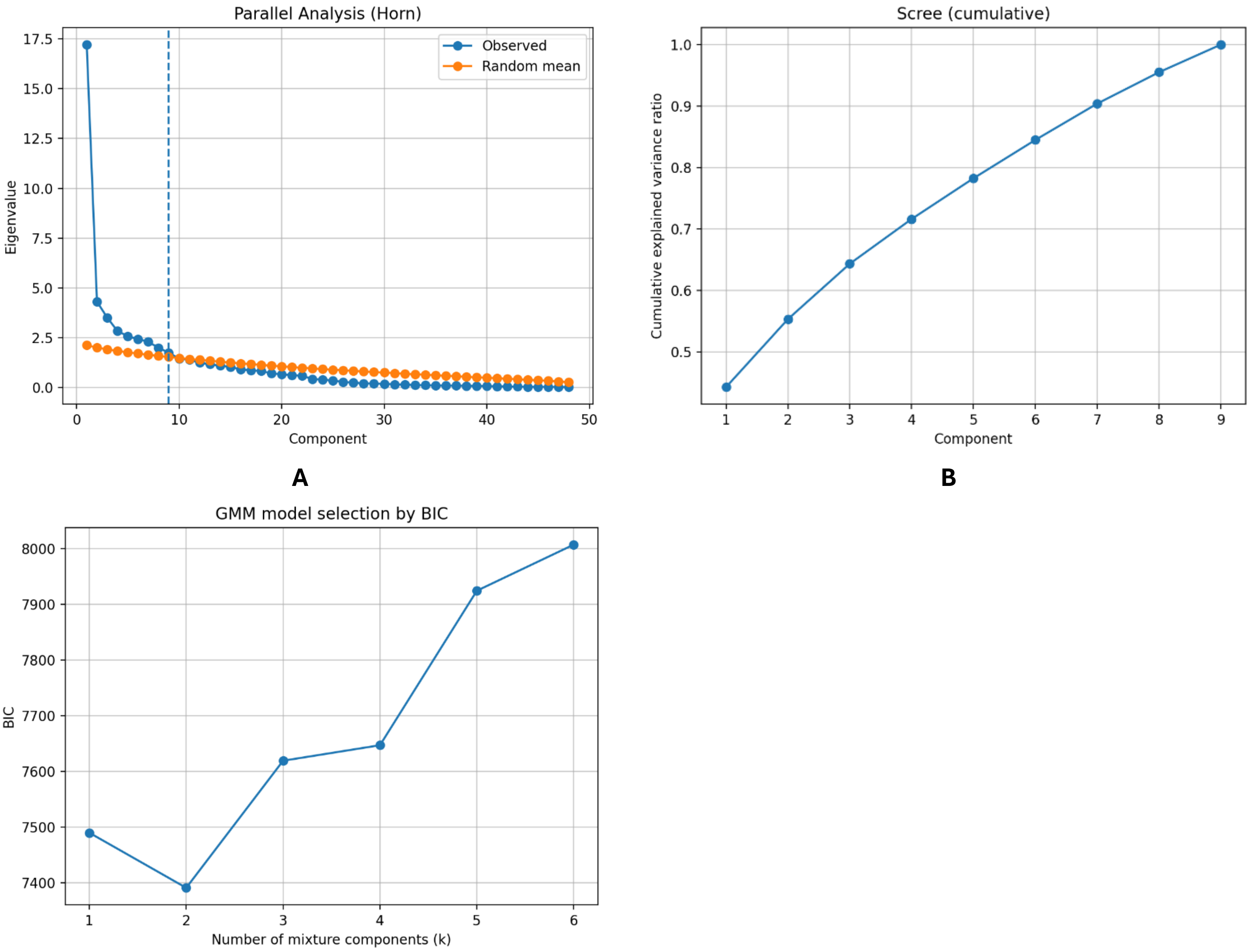

C

Figure S1. Factor retention and mixture selection. (A) Horn's parallel analysis: observed eigenvalues (blue) versus random-data mean (orange). Components with observed eigenvalues greater than the random mean were 1–9 (retained); the crossover occurs at component 10. (B) The cumulative explained variance of the first 1–9 components was 0.43, 0.54, 0.65, 0.74, 0.81, 0.86, 0.90, 0.95, 1.00, respectively. (C) GMM model selection by Bayesian Information Criterion (BIC) across k=1–6 mixture components; BIC was lowest at k=2. Approximate BIC values (from the plotted series): 7,480 (k=1), 7,400 (k=2, minimum), 7,610 (k=3), 7,640 (k=4), 7,930 (k=5), 8,010 (k=6).

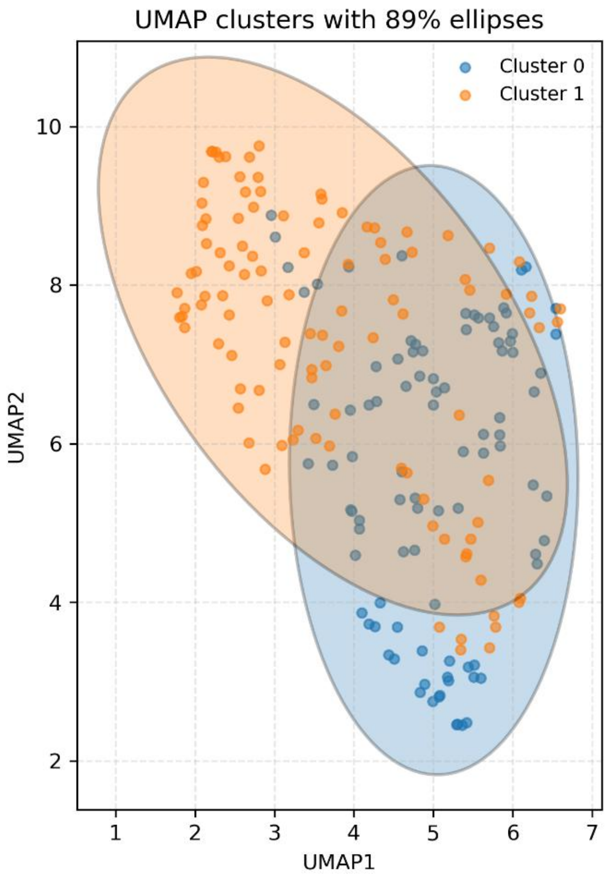

Figure S2. UMAP Embedding. silhouette = 0.174, Calinski–Harabasz = 50.024, and Davies–Bouldin = 1.797. In the UMAP embedding, the between-cluster centroid distance = 2.115 and Bhattacharyya coefficient = 0.291

Table S1. Sample size adequacy for logistic regression endpoints. For each endpoint (insomnia, excessive daytime sleepiness, and extreme chronotype), we report the total sample size, number of events, event proportion, and events-per-variable (EPV) relative to the nine retained factors. EPV values ≥10 are typically considered adequate for logistic regression; lower values indicate limited adequacy.

Outcome	Total Numbers	Number Events	Event Rate	EPV	Non EPV
**Insomnia (ISI≥15)**	200	85	0.425	9.444	12.778
**EDS (ESS≥10)**	200	57	0.285	6.333	15.889
**Extreme Circadian (rMEQ≤7 or ≥22)**	200	30	0.15	3.333	18.889

Table S2. Factor Scores and Severity Symptoms Correlation**.** Spearman correlations between latent symptom factors (F1–F9) and validated sleep measures: composite symptom index (CSI), Insomnia Severity Index (ISI), Epworth Sleepiness Scale (ESS), Pittsburgh Sleep Quality Index (PSQI), and reduced Morningness–Eveningness Questionnaire (rMEQ). Values reported are correlation coefficients (ϱ) with corresponding p-values. Positive correlations indicate higher factor scores associated with worse symptom severity, while negative correlations indicate inverse associations.

	CSI		ESS		ISI		PSQI		rMEQ	
**Factor**	ρ	**P-value**	ρ	**P-value**	ρ	**P-value**	ρ	**P-value**	ρ	**P-value**
**Factor 1**	0.4533	1.59E-11	0.3181	4.43E-06	0.7268	3.82E-34	-0.4547	1.34E-11	-0.2127	0.0025
**Factor 2**	-0.0416	0.5588	0.0826	0.2448	-0.0177	0.8041	-0.1058	0.1361	0.0988	0.1640
**Factor 3**	0.2166	0.0021	-0.1581	0.0253	0.5798	2.36E-19	-0.1176	0.0973	-0.1709	0.0155
**Factor 4**	0.3022	1.37E-05	0.2051	0.0036	0.3595	1.72E-07	-0.1530	0.0305	-0.1504	0.0336
**Factor 5**	-0.1394	0.0490	-0.2196	0.0018	-0.3037	1.24E-05	0.3125	6.62E-06	0.1961	0.0054
**Factor 6**	-0.1817	0.0100	-0.1173	0.0981	-0.4330	1.51E-10	0.3600	1.64E-07	0.2058	0.0035
**Factor 7**	-0.0120	0.8661	-0.1473	0.0374	0.1843	0.0090	-0.0812	0.2528	-0.1681	0.0173
**Factor 8**	0.1711	0.0154	0.1381	0.0512	0.2850	4.30E-05	-0.2503	0.0004	-0.0740	0.2980
**Factor 9**	0.3850	1.81E-08	0.4882	2.26E-13	0.4751	1.18E-12	-0.4785	7.66E-13	-0.2676	0.0001

Table S3. Complete rotated factor loading matrix. Varimax-rotated loadings for all symptom features (rows) across the nine retained factors (columns). Values represent standardized loadings of each feature on the latent factors. This table provides the full factor–feature mapping underlying the dimensionality reduction analysis.

Variable	F1	F2	F3	F4	F5	F6	F7	F8	F9
Age (years)	-0.038	0.100	0.101	-0.133	0.023	0.011	-0.106	-0.003	-0.047
Gender (Male)	-0.012	0.002	-0.031	0.026	0.032	0.058	-0.002	-0.022	0.019
BMI	-0.065	-0.059	-0.026	-0.112	0.342	-0.116	-0.067	0.068	0.026
Race [0-5]	-0.016	0.002	0.003	0.953	0.010	-0.014	-0.010	-0.008	-0.024
ESS score	0.115	0.042	-0.204	0.071	0.072	-0.012	-0.095	-0.052	0.203
ISI score	0.188	-0.022	0.156	0.096	-0.002	0.013	0.005	-0.063	0.061
No sleep problem (yes)	-0.020	0.000	-0.002	-0.001	-0.010	-0.002	0.012	0.013	0.000
Snoring (yes)	-0.002	-0.002	-0.013	-0.021	0.106	0.028	-0.019	-0.014	-0.046
Breathing stops at night (yes)	-0.010	-0.002	0.001	-0.020	0.073	0.014	-0.002	0.033	-0.039
Daytime sleepiness [0-5]	0.069	-0.010	-0.046	-0.015	0.026	0.003	-0.018	0.043	0.069
Unrefreshing sleep [0-5]	0.087	0.003	-0.003	0.026	-0.012	-0.014	0.004	-0.012	0.017
Difficulty falling asleep [0-5]	0.050	0.005	0.112	0.011	-0.028	0.032	-0.012	-0.012	-0.032
Difficulty staying asleep [0-5]	0.036	-0.012	0.081	0.004	-0.026	0.061	-0.027	-0.023	0.004
Irregular sleep schedule (yes)	0.027	-0.002	0.072	0.003	-0.005	0.042	0.012	-0.019	0.011
Sleep behavior (talk/walk/etc) [0-5]	0.029	-0.019	-0.005	0.002	0.005	0.008	0.020	-0.013	-0.017
Nightmares (yes)	0.036	-0.002	-0.014	0.019	0.033	0.014	0.024	0.000	-0.007
Acting out dreams (yes)	0.000	-0.008	-0.005	0.013	0.019	0.028	-0.008	-0.020	-0.003
Restless legs or leg discomfort (yes)	0.029	-0.001	0.014	0.040	0.022	0.022	0.024	0.007	0.000
Muscle weakness when surprised (yes)	0.020	0.007	0.011	0.010	0.008	0.019	-0.006	-0.011	-0.001
Other sleep problem (yes)	0.025	-0.005	-0.005	-0.017	-0.006	0.000	0.013	0.003	-0.016
Bruxism (teeth grinding) (yes)	0.026	0.030	-0.014	0.016	-0.002	0.008	-0.011	-0.055	0.031
Parasomnia (yes)	-0.004	-0.026	0.006	0.007	0.046	0.041	-0.033	-0.020	0.015
Breathing symptoms (yes)	0.021	0.027	-0.046	0.010	0.071	0.016	-0.001	-0.020	-0.030
RLS likelihood (binary) (yes)	0.018	0.026	-0.008	0.001	0.037	-0.002	0.032	0.006	-0.006
FOSQ Score	-0.050	0.002	0.041	0.002	-0.026	-0.031	0.004	0.019	-0.069

rMEQ Total Score	0.017	0.061	-0.038	-0.016	-0.045	-0.063	-0.058	0.086	-0.131
MAP score	0.048	0.094	0.002	-0.034	-0.006	0.033	-0.098	-0.081	-0.015
Cortisol level	0.111	-0.147	-0.123	-0.057	-0.055	0.026	0.081	0.131	-0.227
Unrefreshing sleep (self-report) [0-5]	0.567	0.029	-0.069	-0.064	-0.055	-0.034	0.087	0.008	-0.094
Difficulty sleeping (self-report) [0-5]	0.488	-0.018	0.362	-0.016	-0.002	-0.002	-0.067	-0.131	-0.157
Daytime sleepiness (self-report) [0-5]	0.430	0.011	-0.217	-0.034	0.079	-0.085	-0.055	-0.084	0.326
Hospitalized for COVID	0.010	0.004	0.006	0.002	0.009	-0.014	-0.004	0.001	-0.002
COVID vaccination status (yes)	0.001	-0.020	-0.001	-0.002	-0.028	0.006	-0.026	0.015	-0.015
Sleep symptom prevalence (yes)	0.014	0.017	-0.009	-0.009	0.020	0.003	-0.008	0.008	-0.014
Headaches [0-5]	0.032	-0.109	-0.028	-0.069	0.106	0.586	-0.045	0.064	-0.043
Nasal congestion [0-5]	0.035	0.196	0.050	-0.037	0.207	0.016	0.222	-0.134	0.017
Fatigue [0-5]	0.179	-0.004	-0.068	0.001	0.016	0.022	-0.073	0.444	0.011
Brain fog [0-5]	0.216	0.065	-0.167	0.109	0.114	0.094	-0.017	0.302	-0.013
Unrefreshing sleep (symptom) [0-5]	0.262	-0.081	0.105	0.063	0.044	0.097	0.070	0.109	0.068
Insomnia Long COVID Clinic [0-5]	-0.009	0.003	0.793	0.008	0.035	-0.012	-0.007	0.071	0.079
Lethargy [0-5]	-0.039	-0.010	0.071	0.043	-0.068	-0.012	0.001	0.037	0.800
Post-exertional malaise [0-5]	-0.079	0.033	0.092	-0.022	0.001	-0.041	0.023	0.759	0.034
Change in smell [0-5]	0.001	0.660	0.016	0.021	-0.006	0.026	0.008	0.024	-0.008
Change in taste [0-5]	0.010	0.658	-0.018	-0.014	-0.023	0.006	-0.006	0.033	-0.008
Anxiety or depression (yes)	0.005	0.000	-0.007	-0.010	0.000	-0.015	0.922	0.006	-0.001
Cough [0-5]	-0.029	0.043	0.016	-0.023	0.415	-0.017	-0.037	-0.097	0.044
Shortness of breath [0-5]	0.001	-0.022	0.036	0.035	0.749	-0.005	0.013	0.021	-0.080
Lightheadedness [0-5]	-0.017	-0.063	0.006	0.072	0.125	0.233	0.138	0.035	0.249
Gastrointestinal symptoms [0-5]	-0.043	0.080	0.000	0.001	-0.075	0.732	-0.010	-0.069	0.000

Table S4. Between-cluster comparisons of demographic and clinical features. Summary of continuous and categorical variables compared between Cluster 0 (n=95) and Cluster 1 (n=105). Continuous variables are reported as mean (median) by cluster, with p-values from Mann–Whitney U tests. Categorical variables are shown as counts (percentages) with p-values from χ^2 or Fisher's exact tests, as appropriate. Results are presented for descriptive purposes; full variable-by-variable comparisons are included in the table.

Variable	Level	P-value	Cluster 0 (n=95)	Cluster 1 (n=105)
**Age [years]**	-	0.940499	47.28 (47.00)	47.01 (48.00)
**BMI**	-	0.000115417	24.78 (23.63)	28.58 (26.58)
**Gender [males]**	Male	0.068562704	57 (60.0%)	77 (73.3%)
**Race**	White	0.407088906	62 (65.3%)	77 (73.3%)
	Black	0.407088906	2 (2.1%)	4 (3.8%)
	Native American	0.407088906	1 (1.1%)	2 (1.9%)
	Asian	0.407088906	21 (22.1%)	13 (12.4%)
	Pacific Islander	0.407088906	1 (1.1%)	0 (0.0%)
	Multiple Races	0.407088906	8 (8.4%)	9 (8.6%)
**ESS score**	-	0.030281569	6.19 (5.00)	7.62 (6.00)
**ISI score**	-	0.016782793	12.08 (12.00)	14.36 (14.00)
**rMEQ Total Score**	-	0.137640383	15.02 (15.00)	14.07 (14.00)
**MAP score**	-	0.022307781	-1.53 (0.13)	-2.85 (0.24)
**Cortisol level**	-	0.38666999	14.15 (13.00)	13.54 (13.00)
**No Sleep problem**	Yes	0.103899395	5 (5.3%)	1 (1.0%)
**Snoring**	Yes	0.010273887	25 (26.3%)	47 (44.8%)
**Breathing stops at night**	Yes	0.010473883	15 (15.8%)	34 (32.4%)
**Daytime sleepiness**	Yes	0.159296697	57 (60.0%)	74 (70.5%)
**Unrefreshing sleep**	Yes	0.086585297	65 (68.4%)	84 (80.0%)
**Difficulty falling asleep**	Yes	0.117643586	41 (43.2%)	58 (55.2%)
**Difficulty staying asleep**	Yes	0.249575107	53 (55.8%)	68 (64.8%)
**Irregular sleep schedule**	Yes	0.037164507	28 (29.5%)	47 (44.8%)
**Sleep behavior (talk/walk/etc)**	Yes	0.377520652	8 (8.4%)	14 (13.3%)
**Nightmares**	Yes	0.074456462	20 (21.1%)	35 (33.3%)
**Acting out dreams**	Yes	0.382338551	5 (5.3%)	10 (9.5%)
**Restless legs or leg discomfort**	Yes	0.107756199	21 (22.1%)	35 (33.3%)
**Muscle weakness when surprised**	Yes	0.158187769	4 (4.2%)	11 (10.5%)
**Other sleep problem**	Yes	0.652496115	4 (4.2%)	7 (6.7%)
**Bruxism (teeth grinding)**	Yes	1	35 (36.8%)	38 (36.2%)
**Parasomnia**	Yes	0.022596341	8 (8.4%)	22 (21.0%)
**Breathing symptoms**	Yes	0.003729465	44 (46.3%)	71 (67.6%)
**RLS likelihood (binary)**	Yes	0.025711168	16 (16.8%)	33 (31.4%)
**FOSQ Binary**	Yes	0.009233832	48 (50.5%)	33 (31.4%)
**Hospitalized for COVID**	Yes	0.336009768	4 (4.2%)	9 (8.6%)
**COVID vaccination status**	Yes	0.032751058	89 (93.7%)	87 (82.9%)

**Sleep symptom**	Yes	0.523334801	5 (5.3%)	9 (8.6%)
**Unrefreshing sleep (self-report)**	Never	0.003595649	19 (20.0%)	7 (6.7%)
	Very Mild		4 (4.2%)	5 (4.8%)
	Mild		16 (16.8%)	12 (11.4%)
	Moderate		33 (34.7%)	31 (29.5%)
	Severe		16 (16.8%)	26 (24.8%)
	Very Severe		7 (7.4%)	24 (22.9%)
**Difficulty sleeping (self-report)**	Never	0.026436589	28 (29.5%)	20 (19.0%)
	Very Mild		10 (10.5%)	9 (8.6%)
	Mild		9 (9.5%)	16 (15.2%)
	Moderate		35 (36.8%)	27 (25.7%)
	Severe		6 (6.3%)	17 (16.2%)
	Very Severe		7 (7.4%)	16 (15.2%)
**Daytime sleepiness (self-report)**	Never	0.014115802	23 (24.2%)	13 (12.4%)
	Very Mild		10 (10.5%)	10 (9.5%)
	Mild		14 (14.7%)	12 (11.4%)
	Moderate		33 (34.7%)	30 (28.6%)
	Severe		11 (11.6%)	26 (24.8%)
	Very Severe		4 (4.2%)	14 (13.3%)
**Headaches**	Never	7.95E-07	39 (41.1%)	20 (19.0%)
	Very Mild		8 (8.4%)	13 (12.4%)
	Mild		30 (31.6%)	16 (15.2%)
	Moderate		14 (14.7%)	23 (21.9%)
	Severe		3 (3.2%)	17 (16.2%)
	Very Severe		1 (1.1%)	16 (15.2%)
**Nasal congestion**	Never	0.000106541	56 (58.9%)	34 (32.4%)
	Very Mild		24 (25.3%)	24 (22.9%)
	Mild		10 (10.5%)	17 (16.2%)
	Moderate		5 (5.3%)	21 (20.0%)
	Severe		0 (0.0%)	8 (7.6%)
	Very Severe		0 (0.0%)	1 (1.0%)
**Fatigue**	Never	5.74E-05	9 (9.5%)	2 (1.9%)
	Very Mild		7 (7.4%)	2 (1.9%)
	Mild		14 (14.7%)	6 (5.7%)
	Moderate		22 (23.2%)	27 (25.7%)
	Severe		33 (34.7%)	30 (28.6%)
	Very Severe		10 (10.5%)	38 (36.2%)
**Brain fog**	Never	1.62E-07	20 (21.1%)	4 (3.8%)
	Very Mild		9 (9.5%)	7 (6.7%)
	Mild		12 (12.6%)	16 (15.2%)
	Moderate		36 (37.9%)	20 (19.0%)
	Severe		14 (14.7%)	29 (27.6%)
	Very Severe		4 (4.2%)	29 (27.6%)

**Unrefreshing sleep (symptom)**	Never	6.09E-05	16 (16.8%)	9 (8.6%)
	Very Mild		5 (5.3%)	5 (4.8%)
	Mild		11 (11.6%)	16 (15.2%)
	Moderate		39 (41.1%)	24 (22.9%)
	Severe		20 (21.1%)	20 (19.0%)
	Very Severe		4 (4.2%)	31 (29.5%)
**Insomnia COVID Clinic**	Never	0.000139849	35 (36.8%)	23 (21.9%)
	Very Mild		7 (7.4%)	9 (8.6%)
	Mild		27 (28.4%)	13 (12.4%)
	Moderate		14 (14.7%)	20 (19.0%)
	Severe		9 (9.5%)	20 (19.0%)
	Very Severe		3 (3.2%)	20 (19.0%)
**Lethargy**	Never	0.001659708	27 (28.4%)	20 (19.0%)
	Very Mild		9 (9.5%)	5 (4.8%)
	Mild		14 (14.7%)	10 (9.5%)
	Moderate		30 (31.6%)	24 (22.9%)
	Severe		10 (10.5%)	25 (23.8%)
	Very Severe		5 (5.3%)	21 (20.0%)
**Post-exertional malaise**	Never	1.60E-10	25 (26.3%)	5 (4.8%)
	Very Mild		8 (8.4%)	0 (0.0%)
	Mild		10 (10.5%)	4 (3.8%)
	Moderate		14 (14.7%)	25 (23.8%)
	Severe		35 (36.8%)	35 (33.3%)
	Very Severe		3 (3.2%)	36 (34.3%)
**Change in smell**	Never	3.66E-13	93 (97.9%)	47 (44.8%)
	Very Mild		1 (1.1%)	24 (22.9%)
	Mild		1 (1.1%)	9 (8.6%)
	Moderate		0 (0.0%)	10 (9.5%)
	Severe		0 (0.0%)	10 (9.5%)
	Very Severe		0 (0.0%)	5 (4.8%)
**Change in taste**	Never	4.29E-13	94 (98.9%)	49 (46.7%)
	Very Mild		1 (1.1%)	21 (20.0%)
	Mild		0 (0.0%)	12 (11.4%)
	Moderate		0 (0.0%)	10 (9.5%)
	Severe		0 (0.0%)	7 (6.7%)
	Very Severe		0 (0.0%)	6 (5.7%)
**Anxiety or depression**	Never	0.00608734	58 (61.1%)	40 (38.1%)
	Very Mild		6 (6.3%)	7 (6.7%)
	Mild		11 (11.6%)	13 (12.4%)
	Moderate		13 (13.7%)	21 (20.0%)
	Severe		6 (6.3%)	12 (11.4%)
	Very Severe		1 (1.1%)	12 (11.4%)
**Cough**	Never	1.18E-08	90 (94.7%)	55 (52.4%)

	Very Mild		1 (1.1%)	17 (16.2%)
	Mild		3 (3.2%)	17 (16.2%)
	Moderate		1 (1.1%)	10 (9.5%)
	Severe		0 (0.0%)	2 (1.9%)
	Very Severe		0 (0.0%)	4 (3.8%)
**Shortness of breath**	Never	2.33E-20	86 (90.5%)	24 (22.9%)
	Very Mild		8 (8.4%)	12 (11.4%)
	Mild		1 (1.1%)	20 (19.0%)
	Moderate		0 (0.0%)	28 (26.7%)
	Severe		0 (0.0%)	12 (11.4%)
	Very Severe		0 (0.0%)	9 (8.6%)
**Lightheadedness**	Never	1.17E-05	41 (43.2%)	19 (18.1%)
	Very Mild		14 (14.7%)	16 (15.2%)
	Mild		26 (27.4%)	26 (24.8%)
	Moderate		13 (13.7%)	20 (19.0%)
	Severe		1 (1.1%)	15 (14.3%)
	Very Severe		0 (0.0%)	9 (8.6%)
**Gastrointestinal symptoms**	Never	9.82E-07	47 (49.5%)	26 (24.8%)
	Very Mild		21 (1%)	15 (14.4%)
	Mild		11 (11.6%)	15 (14.3%)
	Moderate		8 (8.4%)	24 (22.9%)
	Severe		6 (6.3%)	17 (16.2%)
	Very Severe		3 (3.2%)	8 (7.6%)
